# The Effects of Lightning: With Special Reference to the Nervous System

**Published:** 1932

**Authors:** Macdonald Critchley

**Affiliations:** Assistant Physician, National Hospital and Junior Neurologist, King's College Hospital, London


### THE EFFECTS OF LIGHTNING: WITH ESPECIAL REFERENCE TO THE NERVOUS SYSTEM.*

BY

MACDONALD CRITCHLEY, M.D., F.R.C.P.,

*Assistant Physician, National Hospital and Junior Neurologist, King's College Hospital, London.*

---

SCIENTISTS and philosophers have interested themselves in the phenomenon of lightning for the past 2,000 years. Since the time of Seneca[1] numerous treatises have appeared on the nature of lightning and upon its effects upon the organism. Prominent amongst these are the monographs of J. A. Dietericus,[2] 1696, J. A. Münnich,[3] 1732, F. J. Baier,[4] 1756, C. F. Hoffmann,[5] 1766, J. G. Biedermann,[6] 1768, and of P. C. Abilgaard,[7] 1771. Among the more recent works special mention may be made of those by C. C. Bonnet,[8] 1859, and F. Sestier,[9] 1866. Especially valuable have been those medical studies of the major lightning disasters. Thus, L. Weber's report[10] on the Schleswig-Holstein disaster of 1878, in which ninety-two people were struck, is among the first scientific studies of the effects of lightning-stroke.

In more recent times we have F. Panse's investigation[11] of the Königstein disaster. On Easter Monday, 1925, twenty-nine tourists were being conducted by a guide over the fortress of Königstein in Saxony. A lightning-flash struck the party, killing outright three and injuring twenty-six others. The services of Dr. Panse were requisitioned in order to study and assess the effects.

* Communicated to the Bristol Medico-Chirurgical Society, on 9th November, 1932.

Numerous isolated case reports are contained within the literature for the past 200 years, detailing the clinical and pathological consequences of lightning accidents. Among the earlier of these we find the account of Professor Richmann, who was killed in his laboratory in Leningrad while studying the charges collected by a lightning-rod on the roof and conveyed to his room.

In this country, owing perhaps to our comparative immunity from severe thunderstorms, only few studies have been made. A. J. Jex-Blake's Goulstonian Lectures [12] for 1913 on "Death by Electric Currents and by Lightning" require particular mention. Within the last year an interesting account of lightning-strokes has been made by H. A. Spencer, [13] who as Medical Officer in the Transvaal has had considerable experience of the many severe storms encountered in that country.

### THE NATURE OF LIGHTNING.

According to G. C. Simpson, [14] lightning comprises an electrical discharge operating at a potential of some thousands of millions of volts. An enormous amperage is generated, amounting to 20,000 to 100,000 amperes. The current is of the alternating type, with a large number of reversals each second. The duration of the electrical discharge is short, amounting to approximately one-thousandth of a second.

### THE EFFECTS OF LIGHTNING.

Of immediate interest are the effects which lightning-stroke may produce in an individual. In view of the layman's interest in this subject, it is strange that so little accurate scientific information obtains as to what precisely occurs in these cases. Boudin's description (1855) of the characteristic effects of lightning as ". . . amazing, protean, contradictory, mysterious," may be more picturesque than accurate, but it does illustrate the inherent difficulties of the problem.

A considerable proportion of lightning accidents prove fatal. The risk is the greater the more direct the lightning impact; the region of the body most affected is of importance, for according to Sestier, who analysed 600 cases, two out of three are killed when the lightning injures the head.

Widespread injuries may be found on the body, resulting not only from the lightning impact but also from the fall. Clothing and boots may be ripped into fragments or even blown completely off the victim. Cadaveric rigidity comes on early and quickly wears off; decomposition sets in unusually soon. The appearance of the corpse soon after the accident may show unusual trance-like or cataleptic postures. An old-time description tells that eight farm labourers were struck while resting at dinner-time under an oak-tree. They appeared, when found, to be still eating. One held a glass, one was carrying a piece of bread to his mouth and a third held a plate. On another occasion a woman was killed while picking a poppy; the body was discovered with the flower still in the hand.

We are just as uncertain as to the cause of death in lightning accidents as with electrical shocks. Experimental evidence does not necessarily apply to human cases. There are two main theories, namely primary heart failure from ventricular fibrillation, or medullary inhibition of respiration. Older views on this problem are of interest. Münnich (1732) believed death was due to a vacuum caused by the

W

lightning which made the blood give up its gases and tear the weaker blood-vessels, especially in the brain and medulla. Hoffmann (1766) was less dogmatic; he suspected "some lesion of the nerves and brain inaccessible to the senses and too delicate to be demonstrable."

Pathological study in fatal lightning accidents reveals numerous gross injuries in addition to burns and skin markings. Fractures of bones of the limbs or of the spine or the skull may be found. Sometimes a limb is severed as cleanly as by surgical amputation. Within the nervous system gross lesions may be noted. Extravasation of blood from the meningeal or from smaller vessels within the medullary substance are described; at times the whole brain has been found diffluent and disorganized as though subjected to the action of high temperatures.

Even in non-fatal cases considerable injury may be inflicted by a lightning-stroke. Among the more immediate of these effects may be mentioned shock and unconsciousness.

In contradistinction to electrical accidents, the victim of lightning experiences little or no sensation when struck. Often he is rendered unconscious without warning, and on recovery can give no account of what has happened. This has been known for centuries, and Pliny, indeed, affirmed that ". . . . the man who sees the lightning and hears the thunder is not the one who is struck." Too much emphasis cannot be laid here, however, for it is possible that a short period of retrograde amnesia may prevent the victim from recalling and describing the events of the stroke, although sensation may have been abundantly present.

At times, however, sensations are experienced prior to unconsciousness, and are remembered afterwards. The usual description is of a flash of light or of colour; at times there is mention of a "rush of wind." Or the patient may feel himself struck a terrific blow. When several are struck simultaneously the victim may remember noticing his companions fall to the ground unconscious, and more than once it has been stated how gradually and slowly the victims seemed to sink to the ground.

Consciousness is almost always lost; the interval between the lightning-stroke and the unconsciousness is usually of extreme shortness. During this state of concussion the patient lies immobile, and shows none of the restlessness, tremor or myoclonic twitchings which may be seen after electrical shock. On the other hand, the condition differs from that in head injury in the severity of respiratory and circulatory disorders. The duration is rarely prolonged beyond an hour or two, and is usually, indeed, considerably shorter. Recovery is fairly abrupt, but may be succeeded by abnormal psychical states which will be described later. Retrograde amnesia usually does not occur.

Various types of disorder may be found by the patient on recovering consciousness. These may be described under three heads, viz. burns, ocular disorders, and neuro-psychiatric symptoms.

According to Spencer burns may be of three types. (1) *Linear burns*, due to the direct effect of the lightning current. These are seen as strips measuring from one-eighth to one inch in width, and extending at times for a foot or more, "as if the victim had been scourged." Sometimes these burns follow the tract of most perspiration, and may outline every crease and wrinkle of the body (Spencer). (2) *Filigree or*

*arborescent burns.* These are well known even to the laity, who from time immemorial have envisaged them as the imprint of surrounding foliage or scenery upon the victim's body through the medium of the lightning-flash. Several quaint examples of these "keraunographic markings,"—or Lichtenberg's flowers, as they were sometimes called—were reported to the British Meteorological Society in 1857 by Poey:—

A man standing opposite a tree which had just been struck by lightning was very much surprised to find on his chest a facsimile of that tree. (Benjamin Franklin, 1786.)

A boy climbed a tree to unnestle a bird; the tree was struck and the boy thrown to the ground. On his chest was plainly imprinted the image of the poplar-tree and the bird in the nest. (Raspail, 1855.)

During September, 1825, lightning fell on the foremast of the brigantine *Il buon Servo* in the Bay of Arriero. A sailor sitting under the mast was struck dead, and on his back was found the imprint of a horseshoe, similar even in size to the original one fixed to the top of the foremast. On another occasion a sailor, standing in the same situation, had on his breast the impress of a number 4·4 with a dot between the two figures. This exactly resembled the 4·4 which was fixed to the extremity of one of the masts. (Orioli.)

Shortly after Poey's communication, the Secretary of the Meteorological Society received the following communication from James B. Shaw on "the photographic effects of lightning":—

Six sheep were killed by lightning in 1812 at Coombe Hay, four miles from the city of Bath. At that time outside the village lay an extensive wood of hazel and detached oak-trees, in the centre of which there was a clearing where the dead sheep were discovered. When the skins were taken from the animals a facsimile of that portion of the surrounding scenery was visible on the inner surface of each skin. "I remember that it caused a great local sensation at the time, and the skins were exhibited at Bath, and I have no doubt there are many living now in that neighbourhood who could vouch for the correctness of this statement. I may add that the small field and the surrounding wood were so familiar to me and my school-fellows, that when the skins were shown to us we at once identified the local scenery so wonderfully represented."

Although Spencer regards this type of burn as of bad prognosis and indicative of a severe stroke from a considerable streak of current, I have known them appear in slight cases of lightning-stroke, and disappear within twenty-four hours.

The third type of cutaneous lesion comprises the *surface burns*, occurring beneath metallic objects worn or carried. Derendinger narrated in 1865 that he was returning home by train when he found that he had lost his pocket-case, a tortoise-shell affair with a silver monogram of two "D's" intertwined. Later he was summoned to attend a man found unconscious under a tree, having been struck by lightning. On the thigh was a burn shaped liked two interlaced "D's." The doctor suggested that his lost case might be in the man's trouser pocket, and so it proved.

Ocular disturbances are not uncommon immediately after a lightning-stroke, and may even appear after some interval of time. Among the immediate affections of the eye may be mentioned intense photophobia from conjunctivitis and episcleritis. Retinal œdema and even detachment have been described. Sometimes there is a vasomotor disorder within the globe leading to the appearance of papillœdema. Ocular palsies, of a transient nature, are also on record. Among the sequelæ of lightning-stroke two may be mentioned, namely cataract and optic atrophy. The former, although actually not a frequent occurrence, has been known for a great many years; thus we read in Garcaeus: ". . . *Humorem cristallinum oculorum exsiccat et liquefacit, quo exsiccato aut liquefacto, cecitatem*

*sequi necesse est.*" Disorders of the oculo-sympathetic are not unknown, leading to permanent pupillary disorders (mydriasis, aniscoria, loss of light reflex, pupillary myotonia).

Immediately on recovering consciousness the patient may complain of severe pains in the trunk or limbs, or in one segment of the body only. These pains are usually transient in character. Sympathetic disturbances are not rare, and may persist for some time.

Thus there may develop vasomotor spasms, cyanosis, pallor or œdema of a limb, hyperhidrosis, tachycardia, a peculiar hard œdema of the muscles. Disturbances of the oculo-sympathetic system have already been mentioned.

Curious psychological states have been noted immediately after recovery from lightning concussion. The patient may pass into a state of noisy excitement with restlessness and anxiety; sleep is usually much disturbed on the night following the accident. Panse refers to the curious mental attitude of the survivors of the Königstein disaster. Although their mentality was clear, there was a strange calm or disinterestedness in a few of the victims. In most, however, there was considerable agitation. Soldiers who ran up found the victims extraordinarily excited and frightened, crying aloud for help. Each was thinking of himself, and seemed quite indifferent to the possible fate of his relatives. This stage of excitement gradually abated within the course of the following week.

Perhaps the chief characteristic of the lightning-stroke is the resultant impairment in nerve function. Immediately on recovering consciousness the patient usually notices an immobility and loss of feeling in the legs and lower part of the trunk. Many have the sensation as if there were nothing below the waist. Although it is doubtful whether any victim has been carefully examined from the neurological standpoint, we possess sufficient information to know that actual paraplegia of a flaccid type combined with objective loss of sensation does occur. What the state of the reflexes is we do not yet know. Sphincteric control is apparently not affected. Within the course of the next hour or so (often within a much shorter period) sensation and power return to the limbs. A feeling of numbness, tingling or pins and needles accompanies this return ; pain is rarely complained of. At the end of twelve hours there is, as a rule, no demonstrable affection of power or of sensation, though a sluggishness of the tendon jerks has been noted.

Observations made during this period of paraplegia show clearly that severe vasomotor changes are present. The legs are white or livid, the toes often bluish, and the limbs are deadly cold.

Two patients in my series were sheltering from a heavy storm at the end of July, 1932, beneath a tree in Kennington Park. They were simultaneously struck by a flash of lightning and both immediately lost consciousness. Both regained their senses after a few minutes to find that they had lost all power and sensation from the waist downwards. This was confirmed objectively by the doctor at the hospital shortly afterwards, and it was noted that the legs and feet were quite cold and bluish-white " . . . as if going in for gangrene." No objective neurological abnormalities were present when I examined them a few days later.

The paralysing effect of lightning is borne out in a quaint account which I recently came across of the affidavit of one Joseph Lockier made on 30th September, 1806. The pamphlet narrates how Lockier, a servant to Mr. Batchelor, a publican of Monckton Farleigh, near Bath, was struck insensible during a thunderstorm between six and seven in the evening of 19th August, 1806. His senses were restored gradually and imperfectly at first, so that at first he was barely capable of perceiving and distinguishing surrounding objects. He felt excessively cold, "the hands and feet swelled, the power of the lower extremities totally gone and those of the arms much impaired." He was totally incapable of articulating or of uttering any sound whatever, though he frequently attempted to. According to the pamphlet, the patient remained in an exhausted and enfeebled state for about fifteen days, during which time he dragged himself through the woods, subsisting entirely on rainwater and grass. Some time in the commencement of September he heard people in the wood with dogs and guns: he called feebly for assistance, but they thought him an impostor and went away. Eventually he was rescued by four men who bore him off in a cart, his limbs being still paralysed. The carters described his limbs "as flaccid and looking like wasted woman's hands." He could hardly articulate ". . . only in a whisper." He could not raise his arm or support it to have his pulse felt. After a little nourishment his pulse rate increased from ten beats per minute to twenty, the upper extremities became warm, but the legs remained cold, "the toes having the appearance of mortification." He was taken to Mr. Mason, surgeon, of Batheaston, whence he was transferred to the Bath Dispensary. There amputation of the toes was carried out. His case aroused considerable local interest among the medical staff, and a testimony as to the findings was drawn up and signed by Drs. Moodie, Murray and Crawford (physicians), Messrs. Creaser and White (surgeons), on 17th November, 1806.

Although the restoration of function is usually complete, one not infrequently hears the story that transient dysæsthesiæ reappear in the legs with each succeeding thunderstorm. One or two examples of these "psycho-recidives" may be quoted :—

A woman, while ironing, was stunned by a vivid flash of lightning and fell unconscious. Afterwards she felt a creeping sensation and numbness, particularly in one arm. This always increased when the air was charged with electricity, and she was always nauseated before a thunderstorm. (Macdonald, 1886.)

A neuropathic woman of thirty-eight was struck by lightning and her two children killed. For four days she was unconscious and afterwards found a left-sided hemiplegia and hemi-anæsthesia. In three weeks she recovered fully. Two days later, during a thunderstorm, she had a second attack. A third took place three years later under similar circumstances. (Camby, 1894.)

A man hit by lightning was able to resume work two days later. Since then with every succeeding storm he would develop tremors and internal agitation. One day, while working on a building during a storm, he was so overcome with tremor that he fell off the scaffolding and was killed. (Forster.)

Analogous cases have been described by Diener, Minonzio, Gerhardt, Reichl and Panse.

The nature of the lightning paralyses is conjectural. We do not know whether the seat of the dysfunction lies in the spinal cord, in the peripheral nerves, or in the autonomic nervous system, or even, indeed, whether structural changes exist at all. Many incline to the view that the paralytic symptoms are psychogenic in type, emphasizing the abrupt onset after a dramatic and catastrophic cataclysm, their transient nature, and more expecially their re-appearance under analagous circumstances, on subsequent occasions.

Most neurologists are unwilling, however, to subscribe to this conception of a hysterical basis, and prefer to reserve their opinion as to the nature. Some have invoked a hypothetical "specific" paralysing effect of lightning, organic in type. Schuster, for instance, wrote: "The lightning palsies are specific of electric origin; they represent a symptom which is pathognomonic for lightning." Charcot[15] coined the word "keraunoparalysis" to describe this affection.

Even the psycho-recidives may have a foundation on other than psychological factors: for Hoche and Reichl report a case in which a severe red œdema of an arm always returned a day or two before a thunderstorm.

Hysterical manifestations undoubtedly occur frequently after lightning, and the literature is full of instances. Blindness, deafness and loss of speech are the commonest psychogenic sequelæ, though here again organic disease may be present in some degree in the affected organ. Thus, some conjunctival or uveal damage may lead to intense lachrymation and photophobia, which in turn are succeeded by a psychogenic disorder of function.

Choosing at random from the literature cases of hysteria after lightning-stroke, we may quote the following:—

Daniel Brown, a sailor on H.M.S. *Cambrian*, was struck both blind and dumb by a lightning-stroke while at sea in 1799. (Godfrey, 1822.)

A man of forty-five was struck helpless by a lightning-flash. He was not unconscious, but complained of great pain in the eyes; for seven months he was "blind" from spasm of the eyelids. When finally the lids were forced open he cried out in ecstasy that he saw the light, but added that it gave him such acute pain that it could not longer be borne. In course of time the photophobia disappeared. (Ogle, 1858.)

In addition to the usual transient paraplegia after lightning (keraunoparalysis) and the hysterical disorders, it must be admitted that permanent neurological sequelæ may occasionally arise, testifying to a severe structural affection of the nervous system.

In 1908 an inhabitant of Berlin stood with many others sheltering from a storm ; all were struck by lightning, and four were killed. The patient was rendered unconscious and was taken to the Charité Krankenhaus. On recovery, he was paralysed in all four limbs and was aphasic ; numerous burns were present. He remained in hospital for nine months, during which time the speech returned but the paralysed muscles began to waste. In 1931 I was permitted to examine this man at the Hufeland Hospital, Berlin, through the kindness of Professor Schuster, who has courteously permitted me to quote the case. Pupillary inequality and facial asymmetry were found ; conjugate ocular movements were defective to the left. Arms and legs were wasted, especially distally, and electrical reactions of degeneration was found. The tendon reflexes were sluggish or absent. There was a peripheral distribution of sensory loss over the arms and legs.

Somewhat analogous cases have been reported by Porot,[16] who described a lightning-stroke which hit a group of Serbian soldiers resting under some trees on the Macedonian front in 1916. Three developed neurological sequelæ. One, seen seven months after the accident, showed a spastic paraplegia, with diffuse wasting of the lower limbs and a left-sided Babinski response. There was loss of sensation as high as the seventh thoracic segment. A second showed after six months universal tremor, diffuse muscular atrophy, sluggish ankle jerks, patchy sensory impairment over the lower extremities. The third developed a paraplegia suddenly twenty-four hours after the accident ; for one month he was totally helpless. Seen fourteen months later, a spastic paraparesis with pyramidal affection was found, and also a left-sided cataract.

Urechia[17] has reported a case of a man who was struck by lightning while carrying a gun over the left shoulder. On recovering consciousness he felt severe pains in the chest and upper limbs. Weakness, wasting and sensory impairment were demonstrable in the left arm, together with cyanosis and hyperhidrosis. These signs disappeared at the end of three months. A second patient developed suddenly a nerve-deafness in the left ear, following a lightning-stroke.

Psychological sequelæ — other than the post-traumatic hysterias — are not unknown, and a lightning-stroke may be the precipitating factor in the development of a chronic confusional or depressive psychosis.

The following case illustrates a chronic psycho-neurotic state, with paranoid episodes and with curious vaso-vagal attacks, following a lightning-stroke :—

O.H., aged 36, attends my out-patient department at the National Hospital, Queen Square, on account of vaso-vagal turns, general weakness, depression, sleeplessness and inability to concentrate. Her symptoms started sixteen years ago, when she was struck by lightning. She did not lose consciousness, but sight and hearing momentarily left her and she became stiff all over. No permanent neurological abnormality supervened. She was kept under observation for some months, but she ultimately developed a marked paranoid psychosis with delusions of persecution.

Well-known neurological disease of organic type may at times seem to be precipitated by lightning, just as by electrical accident or any other form of trauma. An excellent example of a lightning accident followed almost immediately by the first symptoms of a disseminated sclerosis, has recently been reported by R. S. Allison.[18]

Finally, one may quote those quaint records of the apparently salutary effect of lightning on the course of intercurrent disease. In this way it resembles any other sudden and dramatic phenomenon. On the night of 8th September, 1915, a German Zeppelin dropped an explosive bomb outside the National Hospital, Queen Square. Although not a pane of glass remained unbroken in the vicinity, not a single person was injured; on the contrary, several patients in the hospital who had been paralysed for months were instantaneously cured. Similar results have been attributed to lightning. Naturally most of the disorders so "cured" are of psychogenic type, but one or two concern chronic maladies of varied and indeterminate kind. A few examples may be quoted from Sestier:—

A woman of 44 years who had been paralysed in arms and legs since the age of 6 was terrified by a furious thunderstorm. At its height her brother knocked on the door, which was locked on the inside. Without waiting to find her crutches she leapt out of bed to admit him, and from that moment was able to move naturally. (Dienerbroeck.)

In 1807 a man who had suffered from left-sided infantile hemiplegia was struck by lightning; on recovering consciousness he regained the use of his affected side, but was thereafter afflicted with deafness.

A clergyman named Winter who had sustained an apoplectic hemiplegia in 1761, was awakened one night about a year later by a terrific clap of thunder. He felt his chest crushed by an enormous weight, but next day his paralysis had completely disappeared. (Wilkinson.)

A chronic arthritic, who for twenty years had been taking the waters at Tunbridge Wells, was instantaneously cured by a lightning-stroke. (Gardane.)

In 1781 lightning struck a small village hospital in the Austrian Tyrol; the next day a hemiplegic was able to get up and walk without assistance. (Troostwyk.)

Scoresby relates that the packet-boat *New York* was struck twice by lightning in the course of nine hours. In a cabin reserved for ladies slept an old man, whose obesity and infirmity rendered him so helpless that he had not walked half a mile in the past three years, and he had not once been on deck since the voyage started. After the second shock he got out of bed, went on the bridge, and thereafter appeared to have the full use of his limbs. He was at first strange in manner, but this aberration soon passed off.

Dr. Eason of Dublin reports a case of a woman afflicted with a scirrhous carcinoma of the breast who was struck by lightning while sitting at a window. The current hit her chest, scorching her dress. Two days after the accident Dr. Hicks found to his amazement that the tumour was much softer and smaller ; a little later it had disappeared altogether.

Lightning was credited with rejuvenating powers during a storm which struck the Church of St. Mark, Roveredo, in 1783. The officiating priest, an old man of eighty-four, was struck, his vestments being burned. But following the accident his strength seemed to be renewed ; he walked with much greater elasticity, and was able to read without spectacles. (Troostwyk.)

### BIBLIOGRAPHY.

[1] Seneca, M. A., *Quaestionum Naturalium Libri Septem.*

[2] Dietericus, J. A., *De fulmine et cognatis tonitru ac fulgure*, Altdorfi, 1696.

[3] Münnich, J. A., *Relatio physica medica*, Halberstadt, 1732.

[4] Baier, F. J., *Oratio de fulminibus literatorum ordini fatalibus*, Altdorfii, 1756.

[5] Hoffmann, C. F., *De morte in fulmine tactis*, Halae Magdeb, 1766.

[6] Biedermann, J. G., *De causis subitae mortis fulmine tactorum*, Lipsiae, 1768.

[7] Abilgaard, P. C., *Soc. med. Havn. collectanea*, 1775, 2, 157.

[8] Bonnet, C. C., *Des Effets de la Foudre sur l'homme*, Paris, 1859.

[9] Sestier, F., *De la Foudre, de ses formes et de ses Effets*, 2 vols., Paris, 1866.

[10] Weber, L., *Berichte über Blitzschläge in der Provinz Schleswig-Holstein*, Kiel, 1885.

[11] Panse, F., " Über Schadigungen des Nervensystems durch Blitzschlag," *Monat. f. Psych. u. Neur.*, 1925, 59, 323.

[12] Jex-Blake, A. J., " Death by Electric Currents and by Electricity," *Brit. Med. Jour.*, 1913, i., 425, 492, 548, 601.

[13] Spencer, H. A. *Lightning, Lightning-stroke and its Treatment*, London, 1932.

[14] Simpson, G. C., " The Nature of Lightning," *Nature*, 1930, 124, 301.

[15] Charcot, J., " Wirkung des Blitzschlages auf das Nervensystem," *Wien. med. Woch.*, 1890, 40, 9, 57 and 104.

[16] Porot, A., " Trois cas simultanés de paraplégies organiques causées par la Foudre," *Rev. neur.*, 1917, 24 (ii.), 13.

[17] Urechia, C. I., " Polynévrites et ŋévroses traumatique après la fulguration," *Paris méd.*, 1930, 1, 333.

[18] Allison, R. S., " Disseminated Sclerosis in North Wales," *Brain*, 1931, 53, 391.

